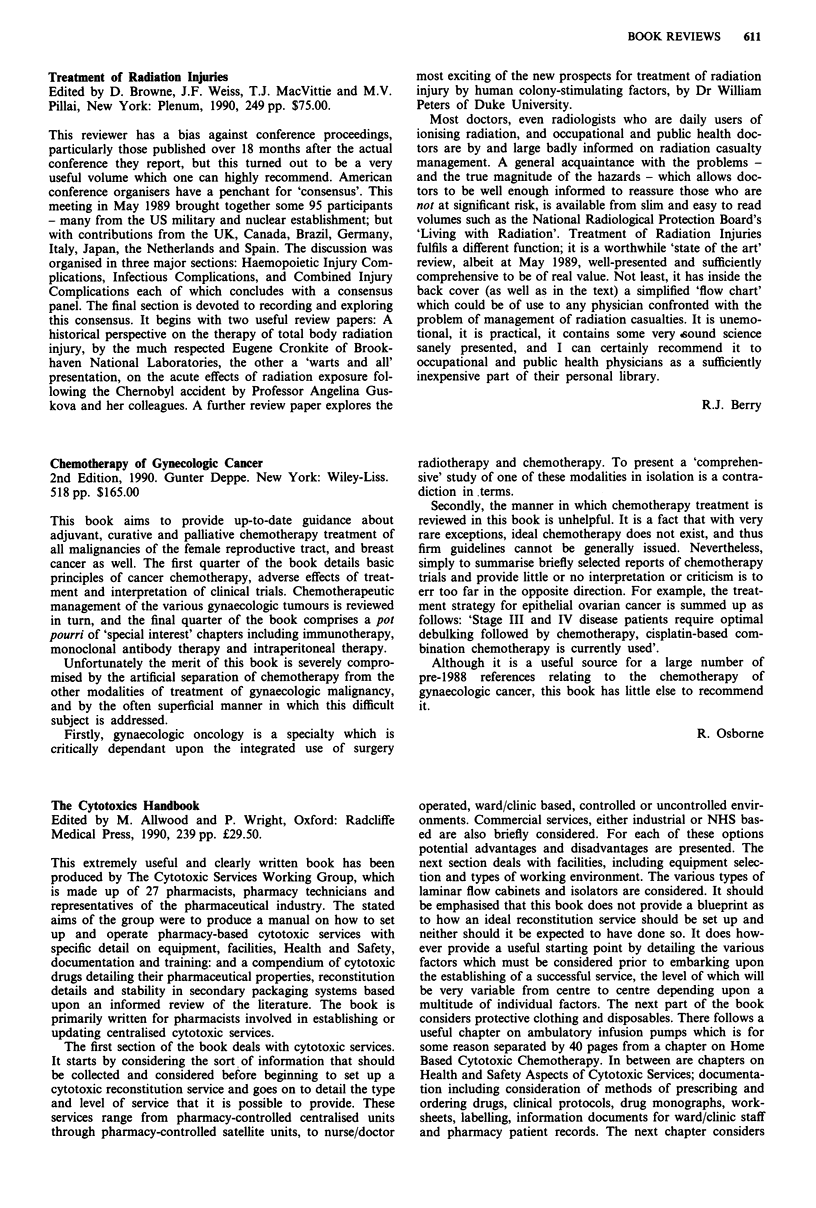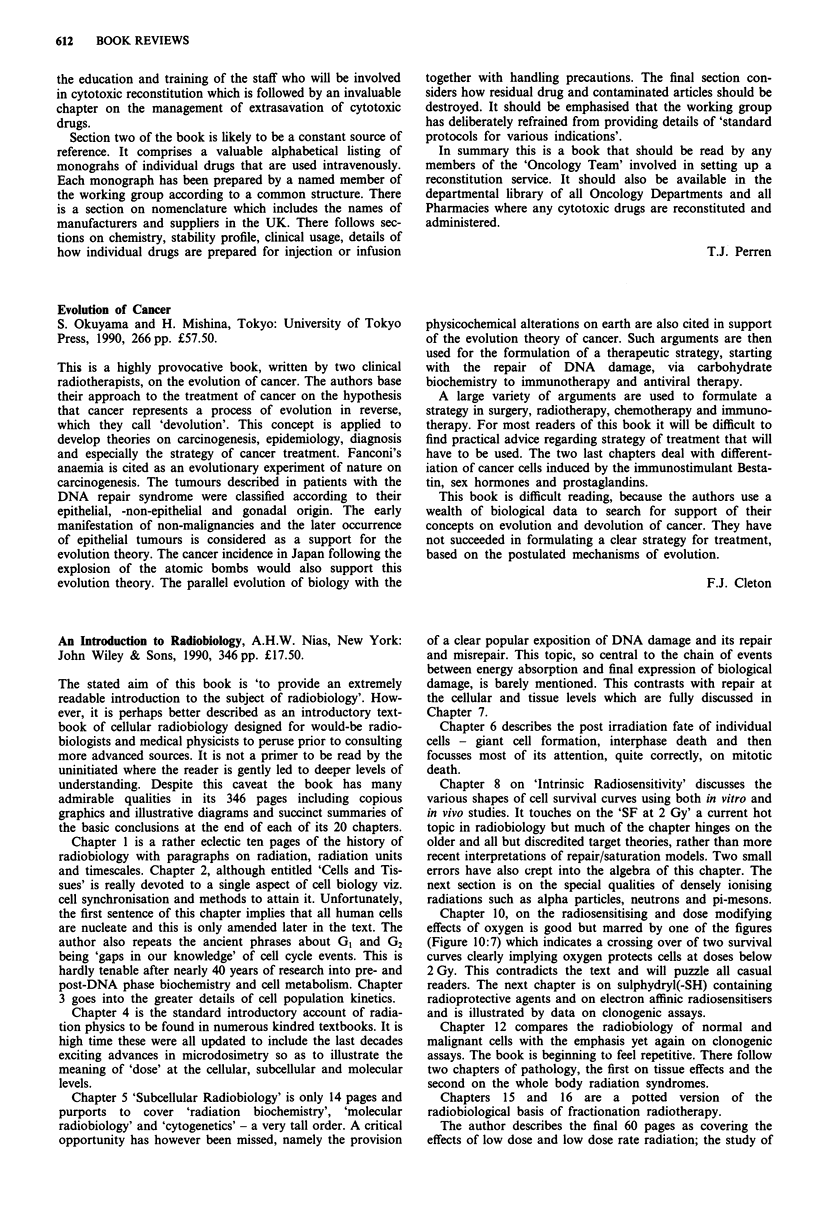# The Cytotoxics Handbook

**Published:** 1991-09

**Authors:** T.J. Perren


**The Cytotoxics Handbook**
Edited by M. Allwood and P. Wright, Oxford: Radcliffe Medical Press, 1990, 239 pp. £29.50.

This extremely useful and clearly written book has been produced by The Cytotoxic Services Working Group, which is made up of 27 pharmacists, pharmacy technicians and representatives of the pharmaceutical industry. The stated aims of the group were to produce a manual on how to set up and operate pharmacy-based cytotoxic services with specific detail on equipment, facilities, Health and Safety, documentation and training: and a compendium of cytotoxic drugs detailing their pharmaceutical properties, reconstitution details and stability in secondary packaging systems based upon an informed review of the literature. The book is primarily written for pharmacists involved in establishing or updating centralised cytotoxic services.

The first section of the book deals with cytotoxic services. It starts by considering the sort of information that should be collected and considered before beginning to set up a cytotoxic reconstitution service and goes on to detail the type and level of service that it is possible to provide. These services range from pharmacy-controlled centralised units through pharmacy-controlled satellite units, to nurse/doctor operated, ward/clinic based, controlled or uncontrolled environments. Commercial services, either industrial or NHS based are also briefly considered. For each of these options potential advantages and disadvantages are presented. The next section deals with facilities, including equipment selection and types of working environment. The various types of laminar flow cabinets and isolators are considered. It should be emphasised that this book does not provide a blueprint as to how an ideal reconstitution service should be set up and neither should it be expected to have done so. It does however provide a useful starting point by detailing the various factors which must be considered prior to embarking upon the establishing of a successful service, the level of which will be very variable from centre to centre depending upon a multitude of individual factors. The next part of the book considers protective clothing and disposables. There follows a useful chapter on ambulatory infusion pumps which is for some reason separated by 40 pages from a chapter on Home Based Cytotoxic Chemotherapy. In between are chapters on Health and Safety Aspects of Cytotoxic Services; documentation including consideration of methods of prescribing and ordering drugs, clinical protocols, drug monographs, worksheets, labelling, information documents for ward/clinic staff and pharmacy patient records. The next chapter considers the education and training of the staff who will be involved in cytotoxic reconstitution which is followed by an invaluable chapter on the management of extrasavation of cytotoxic drugs.

Section two of the book is likely to be a constant source of reference. It comprises a valuable alphabetical listing of monograhs of individual drugs that are used intravenously. Each monograph has been prepared by a named member of the working group according to a common structure. There is a section on nomenclature which includes the names of manufacturers and suppliers in the UK. There follows sections on chemistry, stability profile, clinical usage, details of how individual drugs are prepared for injection or infusion together with handling precautions. The final section considers how residual drug and contaminated articles should be destroyed. It should be emphasised that the working group has deliberately refrained from providing details of 'standard protocols for various indications'.

In summary this is a book that should be read by any members of the 'Oncology Team' involved in setting up a reconstitution service. It should also be available in the departmental library of all Oncology Departments and all Pharmacies where any cytotoxic drugs are reconstituted and administered.

T.J. Perren